# Welfare Assessment of Invertebrates: Adapting the Animal Welfare Assessment Grid (AWAG) for Zoo Decapods and Cephalopods

**DOI:** 10.3390/ani12131675

**Published:** 2022-06-29

**Authors:** Tanya M. Narshi, Danielle Free, William S. M. Justice, Sarah Jayne Smith, Sarah Wolfensohn

**Affiliations:** 1 Institute of Natural Sciences, Imperial College London, London SW7 2AZ, UK; tanya_narshi@outlook.com; 2 Marwell Wildlife, Colden Common, Winchester SO21 1JH, UK; willj@marwell.org.uk (W.S.M.J.); sarahs@marwell.org.uk (S.J.S.); 3 School of Veterinary Medicine, University of Surrey, Guildford GU2 7AL, UK; s.wolfensohn@surrey.ac.uk

**Keywords:** decapod, cephalopod, invertebrates, welfare, sentience, animal welfare assessment grid, quality of life, captive lifetime experience, zoological collections, public aquaria

## Abstract

**Simple Summary:**

The use of decapods (such as lobsters and crabs) and cephalopods (such as octopuses and cuttlefish) by humans for food, experimentation and education (e.g., in zoos and aquariums) is on the increase. Growing evidence that these species have feelings and can experience emotions has highlighted the need for a tool to monitor the welfare of these species in captivity. This study adapted a welfare monitoring tool, the Animal Welfare Assessment Grid, that has been successfully used with a variety of mammal and bird species, for use with decapods and cephalopods. This tool was then trialed at a zoological institution (Marwell Zoo, UK) and, for the first time, a public aquarium (National Marine Aquarium, UK), with the intention of showing how data collected on invertebrates in a zoological environment can be both efficiently and easily applied to implement positive welfare. This study highlights how evaluating the welfare impact of management processes using animal-based indicators can lead to improved welfare outcomes.

**Abstract:**

Consumer demand for invertebrates is on the rise as their numbers in the wild dwindle. However, with the growing conservation efforts of modern zoos and aquariums, and evidence from over 300 studies showing that invertebrates are capable of sentience, public interest, and moral concern for welfare of invertebrates have increased. The challenge for zoos and aquariums is in developing an objective and repeatable method for evaluating welfare that can be applied to invertebrates in zoological collections. Recently introduced into zoological collection management is the Animal Welfare Assessment Grid (AWAG). The AWAG helps to identify negative and positive welfare states, through assessing animal- and environmental-based indicators to make changes that lead to a better quality of life. Originally developed for the assessment of laboratory primates, the system has been successfully adapted to assess a variety of taxa across different environments, facilitated by the development of cloud-based software. In this study, the AWAG has been adapted to assess the cumulative lifetime experience of captive decapods and cephalopods at two different institutions, Marwell Zoo and National Marine Aquarium. This study has provided further evidence that the AWAG is adaptable and demonstrates the first time any objective scoring system has been successfully adapted for use in invertebrates. Through graphical representation, the results show that the AWAG identifies changes in welfare scores that can be attributed to specific events and can be used to predict the future vulnerability of species to welfare changes and suggest alternative management methods. This monitoring tool provides a versatile method of implementing practical welfare monitoring in zoos and aquariums.

## 1. Introduction

There is increasing global awareness of the concept of animal welfare, fueled by social media and resulting in growing public concern as evidenced by the increase in production of meat and dairy alternatives, reduction in the use of fur and leather in fashion, and boycotts of the animal entertainment and tourism industries [1,2,3,4]. These welfare concerns have historically been vertebrate-centric, likely resulting from a combination of: (1) our lack of physical similarity with invertebrates and thus our understanding of and ability to empathise with them; (2) invertebrates’ lack of physical characteristics associated with sentience in vertebrates; (3) the ‘disgust response’; and (4) the idea that small brains result in lack of cognition [5,6]. However, a growing body of evidence supporting the notion that some invertebrates do experience pain and suffering is having a profound effect on how the welfare of these species is considered [7]. 

Following Brexit, where all non-human vertebrates and invertebrates lost legal protection previously afforded to them under EU legislation, the UK government proposed the development of an ‘Animal Welfare (Sentience) Bill’ (from now on referred to as ‘The Bill’). Initally, The Bill planned to recognise all non-human vertebrates as sentient, resulting in all new government policies being required to consider vertebrate animal sentience during their development. Sentience is described by Broom [8] as ‘the ability to feel, perceive and experience’ and is thus inextricably linked with welfare. If an animal is capable of feeling pain and experiencing suffering, then that animal’s welfare can be compromised. Alternatively, their welfare can be positively affected by feelings of happiness, comfort and pleasure. Invertebrates make up 95% of animal life on Earth, and with cephalopod molluscs and decapod crustaceans (from now on referred to as cephalopods and decapods), considered to be the most intelligent and cognitively developed [9], both the public and scientific community argued for the inclusion of these species in The Bill. The extensive evidence gathered by Birch and colleagues [7], supporting sentience in cephalopods and decapods was key to the government’s decision to formally recognise in The Bill ‘any vertebrate other than homo sapiens, any cephalopod mollusc and any decapod crustacean’ [10] as sentient. 

Decapod and Cephalopod use by humans, for food, experimentation and education (e.g., in zoos and) is on the increase [11]. For example, 121,000 tonnes of shellfish (including decapods of various species) landed in UK ports in 2020, an increase from 32,000 tonnes 80 years ago [12]. Worldwide cephalopod catches totaled around 3.6 million tonnes a year in 2017 and 2018, and although this is lower than previous years, this is not due to a lack of demand but reduction in stock, leading to plans for the first octopus farm to be opened by Nueva Pescanova in 2023 [13]. This, coupled with the formal acknowledgement, in the form of The Bill, that cephalopods and decapods are sentient, has identified the need for a welfare monitoring tool for these species in captivity. 

Animal welfare has no singular definition, however, it is generally considered to be ‘the state of the animal as perceived by the animal itself, with regards to its attempts to cope with its environment’ [14], including its perception of both its physical and psychological health. Animal welfare assessments were initially designed for monitoring farm animal welfare but have since been developed for use with companion, laboratory, and exotic animals, and are becoming essential tools for animal carers due to increasing inclusion of the requirement for high welfare standards to conform to laws and legislation [15,16]. To date, most welfare monitoring tools have been mammal-centric with the gradual adaptation for other vertebrate taxa, but with few developments for invertebrates. This is an understandable consequence of previous lack of consensus regarding the sentience of invertebrates. 

The Animal Welfare Assessment Grid (AWAG), a practical animal welfare monitoring tool based on the Five Domains [17], has been successfully trialled with a variety of taxa, including mammals and birds, in a wide range of environments [18,19,20]. Here we are evidencing the success of using the AWAG to objectively assess the welfare of invertebrates, specifically decapods and cephalopods, with the aim to promote the necessity for regular welfare assessment for these species/taxa in captive settings. The number of invertebrates utilised by humans per year vastly outweighs the number of vertebrates, thus the lack of a validated welfare assessment for these could result in untold amounts of suffering. 

## 2. Materials and Methods

### 2.1. Study Subjects 

**Figure 1:**
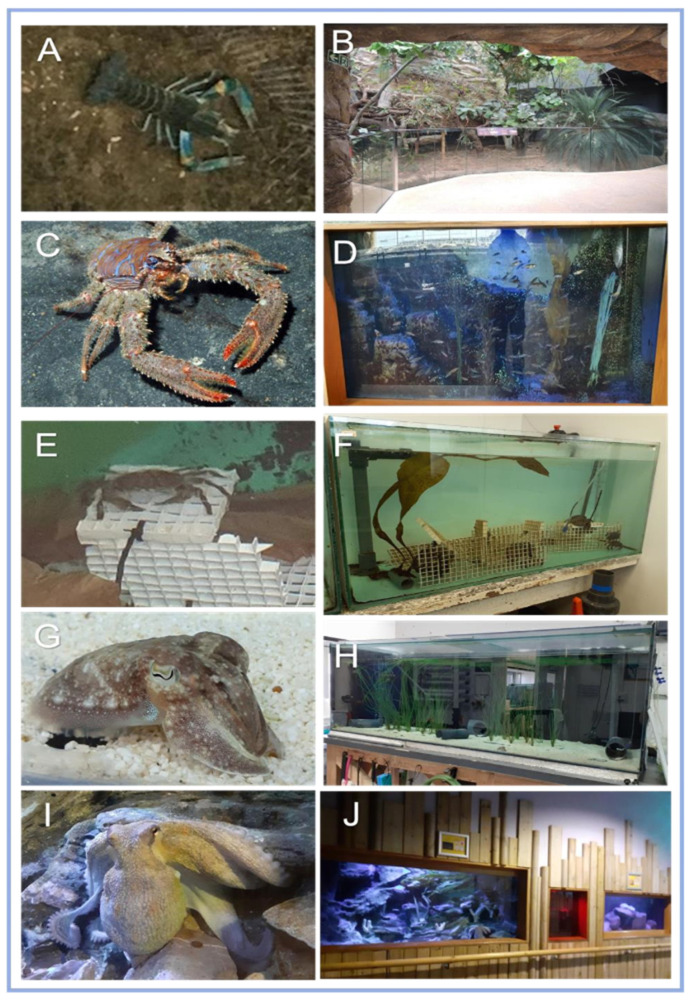
Study subjects. (**A**) Red-clawed crayfish *Cherax quadricarinatus*, housed in (**B**) Marwell Zoo’s tropical house. (**C**) NMA’s squat -lobster *Galathea strigosa* housed in (**D**) Plymouth Sound 6 tank (PS-6). (**E**) NMA’s shore-crab *Carcinus maenas*, initially housed in Temperate Quarantine (TQ) and moved to (**F**) during the trial period. (**G**) NMA’s cuttlefish *Sepia officinalis* housed in (**H**) PS-3 with two male cuttlefish and NMA’s (**I**) common octopus *Octopus vulgaris*, had access to three tanks interconnected by tubes (**J**). (*Photos provided by NMA staff, 2021*).

As the paper aims to ascertain the ease of implementing objective welfare assessment for invertebrates, subjects of this study consisted of three species of decapod; Red-clawed Crayfish *Cherax quadricarinatus* at Marwell Zoo, UK (MZ), comprising approximately 108 individuals (*n* = 108), an individual shore-crab *Carcinus maenas* (*n* = 1) and a squat-lobster *Galathea strigosa* (*n* = 1) both housed at National Marine Aquarium, UK (NMA); and two species of cephalopod; a male cuttlefish *Sepia officinalis* (*n* = 1) and a female common octopus *Octopus vulgaris* (*n* = 1), both housed at NMA (see Figure 1).

### 2.2. Experimental Design

The decapod and cephalopod AWAG scoring templates used in this study were adapted from Wolfensohn et al. [19] and Justice et al. [18]. The AWAGs consist of 19 factors for decapod and 21 factors for cephalopod monitoring, divided into four parameters: physical, psychological, environmental, and procedural (described below). Each factor was scored incrementally from 1 to 10, with 1 being the best possible state relative to the health of the individual and 10 being the most detrimental (see [18,20] for methods). For this study, each factor was chosen using validated indicators of welfare identified from previous studies [21,22,23,24,25,26,27,28,29,30,31,32], and input was provided from zoo and aquarium staff and MZ’s veterinarian on current procedural methods for both taxa (shown in Table 1, Table 2, Table 3 and Table 4).

The researcher, zoo staff, aquarium staff, and volunteers were trained to score the above species (Figure 1) for one hour daily, or three times a week (due to limited staff) as part of an altered management routine, during the trial period: 18 May to 11 August 2021. The scores were recorded with notes detailing events causing score fluctuations.

The AWAG factors were adapted for both individual and group assessment. Group assessments were carried out by randomizing the individuals observed, to reduce bias and to be representative of all individuals within the group. Using overnight video recording, throughout the trial period, the effect of contingent events was also evaluated in the crayfish enclosure (Figure 1B).

#### 2.2.1. Physical Parameters

Four animal-based factors were assessed within the physical parameter class: general condition, activity level, presence of injury/observable clinical signs, and food intake (Table 1). Apart from minor modifications to factor definitions to account for aquatic conditions, the physical parameter class is similar to that scored by Justice et al. [18]. ‘General condition’ was assessed using visual inspection since zoos currently do not weigh their aquatic invertebrates [21], and in group assessments randomized observations were carried out. ‘Activity level’ was monitored to assess any significant changes as a result of stress or illness (omitting any changes resulting from reproductive activity); this proved useful in highlighting any undetected injury or unfavourable environmental changes; in group assessments of decapod invertebrates, the group was assessed as a whole.

In many aquatic invertebrates, it has been shown that feeding frequency is dependent on water quality (including temperature) [26,33]. Therefore, by monitoring food intake in aquatic species, it is possible to infer the presence of insufficient environmental parameters. This was assessed in both individuals and groups by monitoring the amount of food provided and the quantity of food leftover after a feeding period to establish an estimate of food intake at an individual level.

The cephalopod AWAG includes an alternative factor to ‘Presence of Injury’: observable clinical signs (including excessive inking, discolouration, and wounds). Clinical signs are defined by observations that require veterinary consultation; any ‘Observable clinical signs’ will be an indicator of negative welfare [21].

#### 2.2.2. Psychological Parameters

Four animal-based factors were created within the psychological parameter class with the aim of assessing behavioural abnormalities: natural behaviour, abnormal behaviour, response to social disruption, and routine management (Table 2). With little to no veterinary procedures performed on decapods and cephalopods in zoological collections, and a lack of species welfare requirement information, assessing behavioural abnormalities provides an opportunity to monitor animal health, as behaviour can be observed from afar [28]. ‘Abnormal behaviour(s)’ are defined as behaviours that are distressing and maladaptive, examples of these include: erratic/aggressive behaviour, and ‘spinner’ behavior—the inability to control orientation when swimming and location in the water column, as species have a characteristic place in the water tank [21].

The risk of contra-specific ‘Social disruption’ is relatively high in zoos/aquariums [34,35]. This factor was adapted to assess how well the species coped with the presence of staff. ‘Training’ and ‘Response to catching event’ were omitted from this study as neither apply to the study taxa, instead these factors were replaced by ‘Routine management’, a mandatory form of care in zoological settings (including routine handling, husbandry, transport, and tank cleaning) [32]. This factor allows for monitoring and reviewing the degree of disturbance caused by staff.

#### 2.2.3. Environmental Parameters

Seven factors were assessed within the environmental parameter class: water quality, housing/enclosure, group size, enclosure complexity, nutrition, accessibility, and contingent events (Table 3). ‘Water quality’ is a new indicator, added because of its significant value to aquatic animal welfare assessment. Preferred water quality stipulations are species-specific [21]. Monitoring water quality can implement positive welfare by providing means for growth, reproduction, and obtaining resources (including water temperature, salinity, ammonia concentration, dissolved oxygen concentration, and pH levels), and allows for proactive rather than reactive actions as insufficient water quality will cause stress and disease [21].

‘Housing/enclosure’ is species-specific, and considers the size of the enclosure, lighting, shelter, drainage, noise levels, and substrate, and how these allow behaviours, group size, and structure to replicate that of the natural environment. An excessive group size limits resources and shelter availability; this can increase aggressive behaviour and competitive exclusion, as shown in crayfish [36]. Enclosure complexity is monitored to assess the species engagement with all aspects of its environment. Previous studies question whether we should prioritise reducing states of boredom for cognitive species such as octopuses by focusing on enhancing resources in their enclosures [37].

In the UK the primary purpose of zoos and aquariums is to exhibit and preserve animal life for the purpose of conservation, academia, and public interest [33]. Many of the daily activities related to fulfilling this purpose can impact the welfare of the animals held by these institutions. The impact of such activities is scored under ‘Contingent events’. For example, at the time of this study, some aquariums use decapods as educational aids. This may involve housing decapods in rock pools, removing decapods from the water and allowing children to feel their shells. In some cases, time kept out of water can vary, with a guideline of ‘just a few minutes’ [28]—this has been proven to have a detrimental impact on animal welfare [38].

#### 2.2.4. Procedural Parameters

Five factors were assessed within this parameter class: isolation/restraint, effect of intervention, impact of veterinary procedures, change in daily routine, and sedation/anaesthesia (Table 4). Apart from modifications to account for lack of veterinary interventions in both taxa and the aquatic setting, this section did not differ from the factors scored by Justice et al. [18]. Although rare, close-up clinical examinations of these species require manual restraint in the shallows, or out of the water. In group assessments, scores were based on the percentage of individuals that required examination in comparison to the enclosure group size. Sedation is sometimes required for the examination for larger cephalopod species. Sedation/anaesthesia was only assessed in cephalopods as this is rarely used in clinical examination of decapods and veterinary procedures for these species are infrequent [28].

### 2.3. Welfare Analysis

The crayfish at MZ were assessed daily. The shore-crab and cuttlefish were scored daily for 37 days and the squat lobster and common octopus at NMA were scored daily for 36 and 38 days, respectively, throughout the 86-day assessment period. For each species, average daily scores were calculated for all factors within each parameter, using the AWAG software. At the end of each day, the average daily parameter scores were plotted on a radar chart to generate a convex polygon for each day. The area of the convex polygon equated to the cumulative welfare assessment score (CWAS), an overall welfare score. Collectively, the daily CWAS scores were used to present the welfare state over the total trial period. Days on which the assessment was not completed were averaged to show trends in the data.

## 3. Results

### 3.1. Welfare Observations

**Figure 2:**
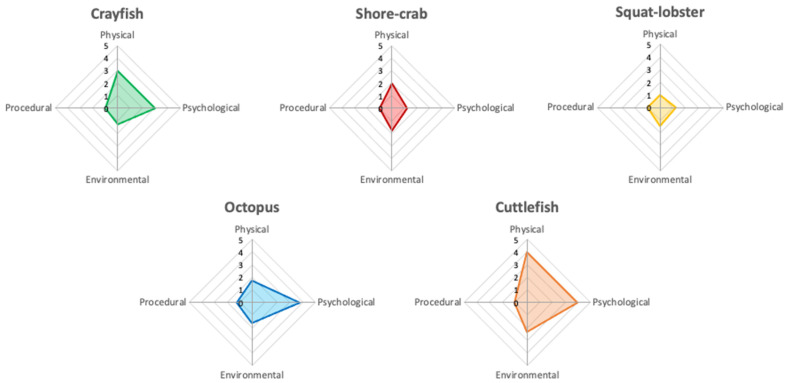
Averaged animal welfare assessment grids of the decapod (**top** row) and cephalopod (**bottom** row) study subjects. The radar charts represent the average scores for physical, psychological, environmental, and procedural parameter class over the study period on a scale from 1 to 10, with 1 being the best possible score and 10 the most detrimental. The axes in the figure are adjusted to increase clarity of the average score for each parameter class for each species. The area of the polygon presented on the radar chart equates to the CWAS value for the complete study period.

Summaries of the AWAG scores, including how individual parameter scores vary over time, daily radar charts and CWAS graphs over the 86-day trial period for each species are shown below. Figure 2 shows the average daily AWAG parameter scores across both taxa for the entire study. General trends for parameter scores remain under a cumulative factor score of 6.00, with high variability across species. There are trends within each taxa group: low (i.e., optimal) average scores for psychological (≤1.20) and procedural (1.00) parameters, and increased (i.e., suboptimal) average scores of physical (≤1.84) and environmental (≤1.51) parameters within the decapod taxa. The cephalopod taxa show low average scores for the environmental (≤1.44) and procedural (≤1.09) parameters but increased physical (≤1.70) and psychological (≤1.73) average parameter scores.

The increased physical parameter scores shown across both taxa similarly stem from a change in general condition because of presence of an injury (‘observable clinical signs’ for the cephalopod taxa). Within the decapods, change in physical condition was attributed to a change in the environment (little to no change occurred with the squat lobster over the trial period). Within the cephalopods, the octopus showed an increase in the average score of the psychological parameter when the physical parameter was affected. The cuttlefish was affected by all but the procedural parameter.

**Figure 3:**
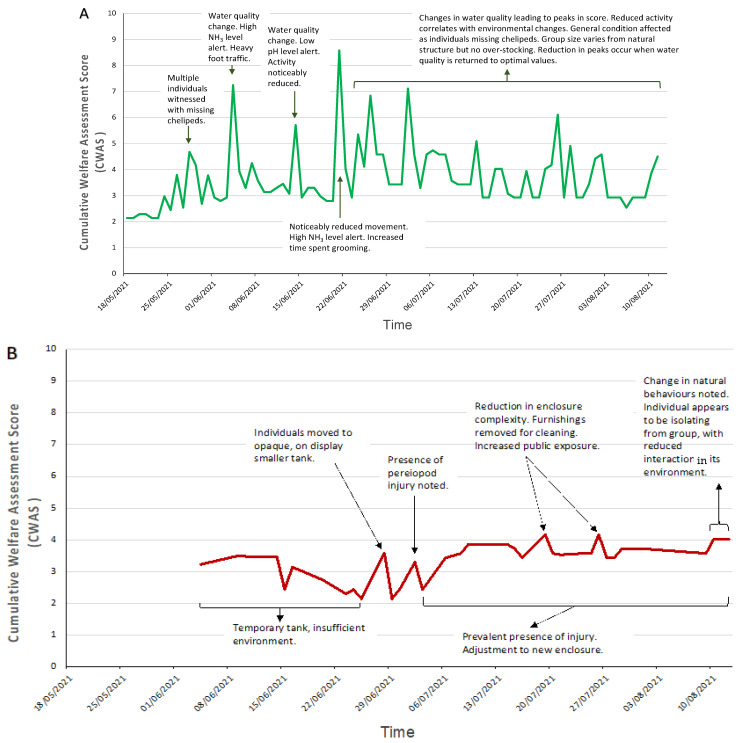

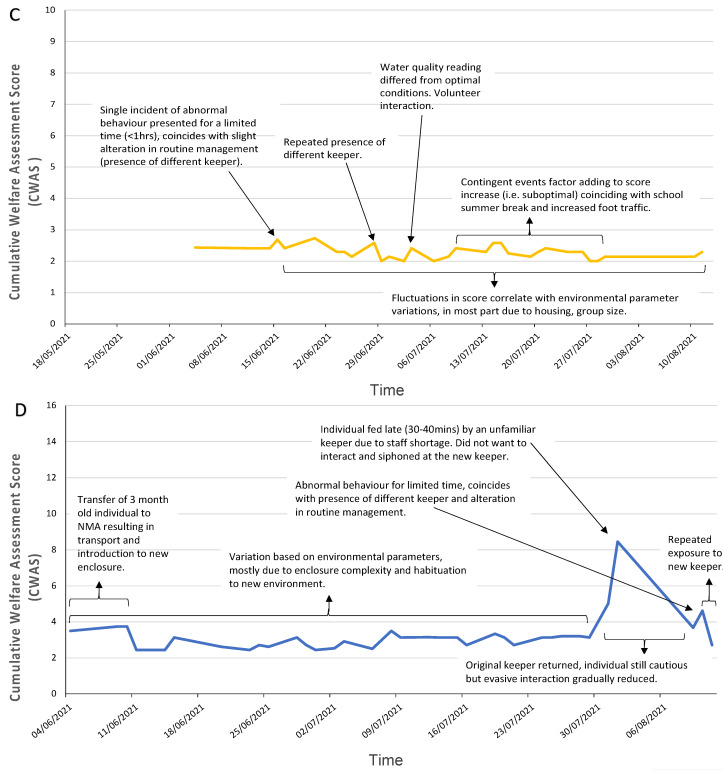

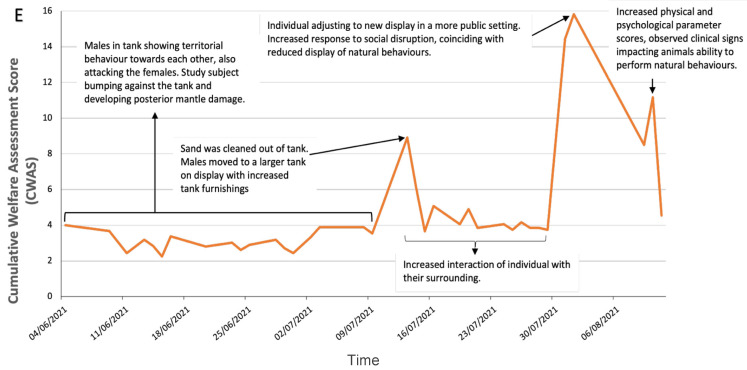
Daily cumulative welfare assessment scores over time for each of the three decapod species (**A**) crayfish, (**B**) shore crab, (**C**) squat lobster, and the two cephalopod species (**D**) common octopus and (**E**) cuttlefish. Annotation of the graphs indicates events that occurred around the time the peak in welfare score was noted (increased value indicates reduction in welfare). A line of general trends is displayed for the days that data were not collected.

The cumulative welfare scores for each species were plotted against time, as shown in Figure 3. Each species displays no similarity in pattern overtime, but similar events occur that result in similar reactions, peaks (i.e., suboptimal) in the welfare score at different intensities.

### 3.2. Decapod Cumulative Welfare Lifetime Experience

**Figure 4:**
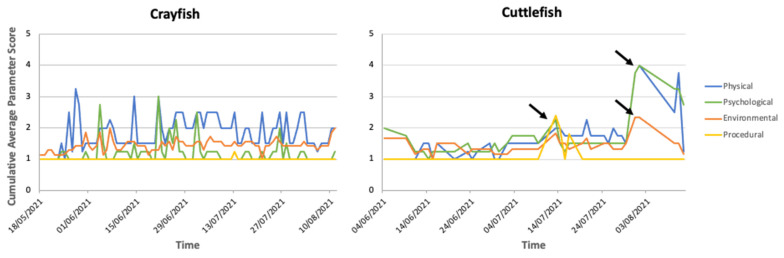
Daily average parameter welfare assessment scores over time for MZ crayfish. Each line presents one of the four assessed parameters: physical, psychological, environmental, procedural, on a scale of 1 to 10. In the figure, the axes are adjusted based on the range of the daily average parameter scores. The black arrows indicate the noticeable parameter changes between two events that incur greater (i.e., suboptimal) welfare scores presented with the cuttlefish assessment.

The CWAS plotted against time for MZs crayfish showed large variation throughout the entirety of the study (mean: 3.68; CWAS range: min 2.18–max 8.58). Figure 3 highlights the events that occurred around the time of the increased scores. Figure 4 shows the breakdown of the CWAS into each of the four parameters. Continual assessment of the crayfish revealed that trends in activity levels and general condition closely matched environmental parameter changes, more specifically water quality and group size changes. The highest average scores presented (7.24, 8.58, 7.12) were a result of the presence of injury and fluctuations in NH_3_ and/or pH levels.

The shore crab also presented considerable variation in CWAS over time (mean: 3.35; CWAS range: min 2.14–max 4.17). The high scores in the data (3.58, 4.17) correlate with environmental parameter changes (due in most part to housing and enclosure complexity), as the individual was moved from an off-show holding area to on-show display tank, and physical parameters (due to presence of injury). The score then remained elevated.

The squat lobster average scores remain close to optimal (mean: 2.29; CWAS range: min 2.00–max 2.73) with all parameters scoring below 2, the data retain a similar shape throughout the assessment.

### 3.3. Cephalopod Cumulative Welfare Lifetime Experience

**Figure 5:**
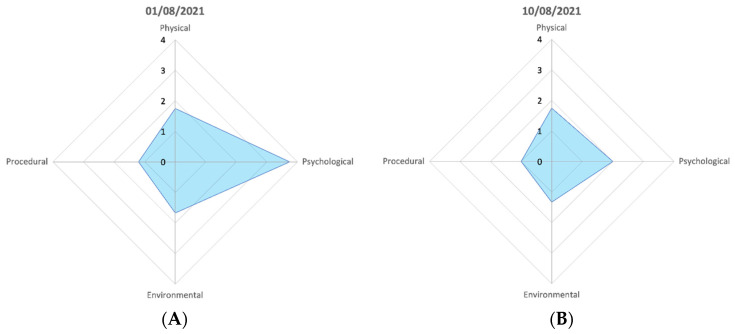
Individual animal welfare assessment grid of the common octopus, the parameter scores of the two greatest peaks in the data. (**A**) shows the parameter scores when a change of keeper and late feeding occurred, (**B**) shows the presence of the same different keeper but a normal feeding time. The shape of the polygon in each is the same but at different magnitudes of change.

Scores were taken on 38 days of the trial period for NMA’s common octopus (mean: 3.23; CWAS range: min 2.44–max 8.46). Figure 3D shows that a peak (8.46) in score is attributed to a change of keeper and late feeding. There is a gradual reduction in the welfare score (3.67) when the original keeper returns. Figure 5 highlights the differences in parameter scores of both events.

Figure 3E shows that the cuttlefish presented higher CWAS scores (mean: 4.7; CWAS range: min 2.44–max 15.82). The data present three substantial peaks (Figure 3B), the first (8.90) is initiated by cleaning of and movement to a larger tank to improve welfare by reducing aggressive behaviour. Mode of transport was not recorded. The second peak (15.82) coincides with an increased public presence. The third peak (11.16) is a result of prolonged presence of posterior mantle burn altering the individual’s behaviour.

## 4. Discussion

The AWAG was successfully adapted to monitor the welfare of the invertebrate species observed in the study. This is the first time this system has been used to assess invertebrates and, as far as the authors are aware, the first time any objective welfare scoring system has been successfully adapted for use in invertebrates. This is a significant result given the need to monitor the welfare of those invertebrate species evidenced as sentient and consequently included within the new Animal Welfare (Sentience) Bill.

Given the relative paucity of information relating to the needs of invertebrate species to maintain good welfare, the study findings also highlight several key areas relating to the welfare of decapods and cephalopods in captivity. This is well illustrated by how the cumulative welfare score for red-clawed crayfish responds to changes in water quality. The trend produced by the AWAG shows various points where increasing scores (indicating a deterioration in welfare) correlate with deviations in water quality parameters outside of the species preferred range. The sensitivity of crayfish to poor water quality is well documented [39]. The behavioural changes observed during the study, including movement into the shallows or out of the water altogether, are consistent with the response of wild crayfish to poor water quality in the environment [30]. This may indicate a negative welfare impact due to changes in water quality. As a resource-based measure it is not a direct measure of the animal’s welfare state, however it is a reasonable proxy given the difficulties in measuring the direct impact of water quality on the physiology of the crayfish.

These observations and the corresponding change in cumulative welfare score help confirm the validity of using the system for welfare monitoring in this species. These findings also suggest another potential use for the AWAG. Given this example shows the AWAG is capable of detecting changes in welfare due to behaviours observed both in captivity and the wild, it may be possible to use the AWAG as a predictive tool for assessing the welfare of wild animals where parameter values (for example, for water quality) are known. This would be an interesting area for further investigation.

The cumulative welfare score for red-clawed crayfish also reflected changes in group size due to the impact of increased aggression within the group. This aggression was likely to be due to competition over limited resources within the captive environment. This provides valuable information for animal managers to help prevent poor welfare within crayfish colonies. The cumulative welfare score may act as an early indicator of increased competition over resources. Management interventions, such as provision of the lacking resource or reduction in group numbers, can then be made before welfare is significantly compromised. This is also a good example of how the AWAG can be used to assess group welfare and supports the findings of previous studies [18]. When assessing large group sizes, a focus on individuals can be both impractical and detract from group level factors (such as the level of competition within the group) which may have a significant positive or negative impact on welfare. This has potential implications for monitoring the welfare of any colony living species, especially where colonies are comprised of large numbers of individuals.

The cumulative welfare scores for the shore crab highlighted the importance of the physical environment to decapod welfare. This is reflected in the Housing and Environmental Complexity factors under the Environment parameter in the AWAG. Both factors are scored relative to the resources available and complexity in the wild. The cumulative welfare score shows a significant difference between the shore crab off-show holding facilities and the on-show display tank. This highlights the difference in environmental complexity when comparing on-show and off-show areas. This difference is a frequent finding in zoos and aquaria and often occurs due to a heavy emphasis on functionality (such as ease of cleaning) in the historical design of off-show areas. Improving the interactive complexity of off-show areas should contribute to improving overall welfare [40]. Interestingly, there was little variation in the cumulative welfare score for the squat lobster. This suggests that where this species is maintained in a consistent, appropriate environment with minimal intervention or change, good welfare can be achieved.

Scheel [41] suggests that octopuses can recognise individual people and may be able to form a relationship with their carers. The findings of this study also support this assertion, as there are clear changes in the cumulative welfare score which correlate with the presence or absence of familiar people. This has implications for when staff changes, or institutional transfers occur as the absence of a familiar carer may be detrimental to welfare. Similarly, the findings also suggest that human interaction with octopus in captivity may be a source of positive welfare. This is consistent with findings in other vertebrate species and perhaps more evidence for sentience [42]. The cumulative welfare scores for cuttlefish reflected aggressive behaviour due to competition over territory. The negative welfare impact seen here, relating to competition over resources, is similar to the observation made for the red-clawed crayfish. This reinforces the importance of ensuring an appropriate level of resource availability for all individuals held in group situations or where an individual perceives competition from a co-terminus species or human carer. Transfer to another enclosure also resulted in a negative welfare impact. This procedure is analogous to the transportation of vertebrate species between different holding areas or institutions, a process previously highlighted as having a negative impact on animal welfare [43]. The welfare impact of transportation of sentient invertebrates would be another area worthy of further investigation and evaluation. Interestingly, an improvement in welfare was noted when the cuttlefish was introduced to the new enclosure, suggesting that activities such as exploration of complex environments may be beneficial to the welfare of this species.

Several limitations of using the AWAG were noted as a result of the study. The number of cephalopods used in the study is too low to be confident that the system works in all cases. However, the information is included here given its importance due to the lack of data for cephalopod welfare assessment. Next, it was noted that the scoring system assumes that signs of fear in response to an aversive stimulus, in this case moving away or hiding from keepers during routine events, suggests a negative welfare impact, when it may be an indicator of better welfare than those that do not move or hide, possibly because of physical impairment, however in the scores recorded such an impact of physical impairment on behaviour was not seen. Additionally, when monitoring at group level some factors had to be estimated for practical reasons, for example food intake. Finally, care needs to be taken not to assume a direct link between cumulative welfare score and environmental parameters for all species, for example some crayfish are capable of tolerating changes in water quality.

As found in previous studies, the findings show that the AWAG can be used in different institutional settings. Although the system has been used in several zoos, the authors believe this is the first trial of the system in a public aquarium. As others have also noted though, the system cannot be used to compare different taxa or institutions due to the difference in factors scored [18]. However, the flexibility of the system allows different sources of information to be used to generate cumulative welfare scores. This, combined with the availability of user-friendly software, makes the AWAG practical to use for continuous monitoring by animal carers.

## 5. Conclusions

To conclude, this study has shown that the AWAG can be successfully adapted and applied to decapods and cephalopods in zoo and aquaria environments, presenting for the first time an objective scoring system for use in invertebrates. The AWAG can easily identify changes in welfare scores that can be attributed to specific events, thus presenting a practical method of assessing the welfare of invertebrates. The importance of this monitoring tool is that it highlights changes in cumulative welfare trends, providing evidence for prompt management interventions that can promote the positive welfare of species in zoological collections.

With invertebrates, insects in particular, being hailed as the ‘food of the future’, and the growing evidence for sentience, it is crucial that we continue to expand our methods for accurately assessing invertebrate welfare.

## Figures and Tables

**Table 1:** Physical parameter scoring for the AWAG assessment (Underlined—factors only assessed in decapods, Italics—factors assessed in cephalopods only).

Score	General Condition (Thoracic Legs Condition and Number, Moulting, Growth, Carapace Integrity: Skin Colour/Texture/Integrity, Abnormal Body Morphology, Eye Condition and Quality of Limbs)	Activity Level (e.g., Foraging, Burrowing, Defending Territory/Food, Aggression)	Presence of Injury (e.g., Damaged Or Missing Limbs, Lameness)	Food Intake	*Observable Clinical Signs (e.g., Bloating, Bulging Eyes, Discolouration and Other Clinical Symptoms)*
1	Optimal condition *(BSC 3)—ideal condition*	Normal	No observable signs of injury	Eating normally (all food placed in the enclosure is eaten before the next feed)	*No observable clinical signs*
2	Slightly under optimal condition with no disruption of activity—all body segments intact *(BSC 2.25/3.75)—Slightly under optimal physical condition*	Increased activity (not caused by normal variation)	Mild signs of injury observed (no missing limbs), or with missing limbs but ability to perform expected behaviours	Food intake slightly lower than normal for one day (small remnants of food in the enclosure when given the next feed)OR animal reported hungry	*Mild clinical signs that show no impact on the animal’s ability to perform expected behaviours. Full recovery expected*
3	Mild signs of injury with only temporary disruption of normal activity < 8 h *(BSC 3.5/2.5)—slightly under optimal physical condition—full recovery in 24 h*	Increased activity or slight reduction in activity (possible cause; environmental factors or reproducing)	Mild signs of injury (no missing limbs) with slight impact on ability to perform expected behaviours	Food intake significantly lower than normal for one day (significant amount of food in the enclosure when the next feed is scheduled) OR reported hungry for 2–3 days	*Mild clinical signs having a short term impact on the animal’s ability to perform expected behaviours. Full recovery expected*
4	Mild signs of injury with only temporary disruption of normal activity < 12 h *(BSC 3.75/2.25)—Moderately under optimal physical condition—full recovery expected*	Increased activity or reduced activity (no direct cause noted)	Mild-moderate signs of injury observed (missing limbs) with medium impact on ability to perform expected behaviours	Food intake slightly lower than normal for 2 days (lower than 80%) OR reported hungry for 4–5 days	*Mild clinical signs having a longer term impact on the animal’s ability to perform expected behaviours. Full recovery expected*
5	Moderate signs of reduced carapace integrity observed - (Antenna and abdominal tail still intact, Majority of pereiopods intact. Chela functional and full use retained.)—Excessive grooming events < 24 h *(BSC 4/2)—Moderately under optimal physical condition.*	Sizeable increase or decrease in activity that shows full recovery not related to courtship	Moderate signs of injury observed (missing limbs) with significant impact on ability to perform expected behaviours	Food intake significantly lower for 2 days (lower than 50%) OR reported hungry for 6–7 days	*Moderate clinical signs having limited impact on the animal’s ability to perform expected behaviours. Full recovery expected*
6	Moderate signs of reduced carapace integrity observed, reduced growth—(Antenna, pereiopods and abdominal tail still intact. Chela functional and full use retained.)—Excessive grooming events observed < 48 h *(BSC 4.25/1.75)—Significantly under optimal physical condition*	Sizeable increase or decrease in activity that shows some recovery not related to courtship	Moderate signs of injury with substantial effect on ability to perform expected behaviours—missing limbs but will fully regeneration	Food intake slightly lower than normal for 3 days (lower than 80%) OR reported hungry for 8–9 days	*Moderate clinical signs having limited impact on the animal’s ability to perform expected behaviours. Recovery unknown.*
7	Significant signs of reduced carapace integrity observed, reduced growth—(Antenna, pereiopods and abdominal tail still intact. Chela functional and full use retained.)—Excessive grooming events observed < 48 h *(BSC 4.5/1.5)—Significantly under optimal physical condition*	Sizeable increase or decrease in activity that is consistent throughout the day	Moderate signs of injury observed with severe prolonged impact on ability to perform expected behaviours—missing limbs with full regeneration but does not regain full use of limb	Food intake significantly lower than normal for 3 days (lower than 50%) OR reported hungry for >9 days	*Moderate clinical signs with medium to long term impact on animal’s ability to perform expected behaviours. Recovery unknown*
8	Markings, significantly poor carapace integrity and missing limbs (potential sign of overstocking), relative size of species smaller than expected, reduced moulting events—has some ability to function *(BSC 4.75/1.25)—Severely under optimal physical condition—Frequent abnormal displays*	Minimal movement or signs of hyperactivity	Severe signs of injury observed with severe prolonged impact on ability to perform expected behaviours—missing limbs with full regeneration but does not regain full use of limb	Not eaten for 3 days	*Severe clinical signs but with short term impact and expected recovery OR moderate to severe signs with long term impact on animal’s ability to perform expected behaviours and little chance of recovery*
9	Markings severely poor carapace integrity and missing limbs (potential sign of overstocking), relative size of species smaller than expected, reduced moulting events, discolouration visible—has little functioning ability *(BSC 5/1)—Severely under optimal physical condition—little chance of recovery*	Lethargy or hyperactivity	Severe or chronic signs of injury observed with life threatening impact on ability to perform expected behaviours—missing limbs with inability to regenerate	Not eaten for 5 days	*Severe or chronic clinical signs that are having serious negative impact on the animal’s ability to perform expected behaviours*
10	Severe poor general condition - discolouration, immobile, no carapace integrity, growth limitation (no longer able to moult) and no sign of reproductive ability *(BSC 0 or 5+)—Entire animal pale and fails to change colour when challenged and swelling*	Complete lethargy (no movement, possible minimal movement when encouraged)	Severe or chronic signs of injury with life-threatening impact on ability to perform expected behaviours—multiple missing limbs without the ability to regenerate. Causing complete recumbency and/or lack of all normal behaviours	Not eaten for 7 days-food in enclosure is untouched	*Severe clinical signs that are rendering the animal recumbant/unable to carry out any expected behaviours*

**Table 2:** Psychological parameter scoring for the AWAG assessment.

Score	Abnormal Behaviour (e.g., Burrowing Behaviour, More Time Spent in Hiding)	Response to Social Disruption	Routine Management	Natural Behaviours (Species Specific, Seen or Fresh Evidence of e.g., Various Modes of Locomotion, Wallowing, Ruminating, Scent Marking, Resting, Feeding, Grooming, etc...)
1	None	Zookeepers/aquarists have no effect on the behaviours displayed, completely habituated	Animal(s) shut off easily and with no intervention required OR has complete access to enclosure	All behaviour expressed is natural as expected in the wild
2	Single incidence of the behaviour observed, able to be distracted	Minimal response to zookeepers/aquarists presence and show no stress, well habituated	Minimally difficult, slight enticement successful	All behaviours expected in captivity have been observed
3	Low frequency, minimal time spent performing behaviour(s), able to be distracted	Moderate response to zookeepers/aquarists and show no stress, well habituated	Moderately difficult, significant enticement required	Animal(s) is displaying a wide range of natural behaviour and no abnormal behaviour
4	Low frequency, some time spent performing behaviour(s), able to be distracted	Noticeable change in behaviour in response to zookeepers/aquarists, slight sign on stress/fear	Moderately difficult, significant enticement required with more than one attempt OR animal(s) left with access	Animal(s) is displaying only a few of the natural behaviours expected, no abnormal behaviour
5	Moderate frequency, some time spent performing, able to be distracted	Distinct change in behaviour in response to zookeepers/aquarists. Moderate stress/	Difficult, animal(s) is slightly reluctant to be shut off, higher intervention required (e.g., herding vocally)	Animal(s) is displaying mostly natural behaviour with/without infrequent unnatural behaviour observed
6	Moderate frequency, considerable time spent performing, difficulty distracting	Noticeably stressed/scared in zookeepers/aquarists presence. With/without mild aggression	Difficult, animal(s) is very reluctant to be shut off, higher intervention required, moderate stress observed (e.g., herding physically)	Animal(s) is displaying mostly natural behaviour with more frequent unnatural behaviour observed
7	High frequency, considerable time spent performing, difficulty distracting	Elevated signs of stress/fear in response to zookeepers/aquarists. With/without moderate aggression	Very difficult, animal(s) is very reluctant to be shut off, intervention required, moderate stress	Ability to display natural behaviour is impinged, increase in unnatural behaviour
8	Higher frequency, considerable time spent performing, disrupts normal routine/behaviour and not able to be distracted	Further elevated signs of stress/fear in response to zookeepers/aquarists. With/without significant aggression	Very difficult, extremely reluctant, high stress levels for prolonged time, more severe intervention required. (e.g., trapping)	Limited natural behaviours observed, more unnatural behaviours observed than natural
9	Very frequent, majority time spent performing, disrupts normal routine/behaviour, not able to be distracted	Severe signs of stress/fear in response to zookeepers/aquarists. With/without severe aggression	Extremely difficult, extreme stress experienced, extreme intervention required	Mostly unnatural behaviour observed, almost complete lack of natural behaviour
10	Constant, all of the animal’s time spent performing behaviour(s), unable to distract, and normal routine/behaviour disrupted	Extremely scared and/or aggressive in response to zookeepers/aquarists with potential to cause danger to themselves and/or the zookeepers/aquarists	Animal(s) is harmed in the process of shutting off and experiences extreme stress	Complete lack of natural behaviour observed, overwhelming abnormal behaviour present

**Table 3:** Environmental parameter scoring for the AWAG assessment (Underlined—factors assessed in decapods only).

Score	Water Quality (Species-Specific, e.g., Water Temperature, Salinity, Ammonia Conc., Dissolved Oxygen Conc., pH)	Housing/Enclosure (Species Specific, e.g., Size, Lighting, Shelter, Noise Levels, Substrate etc.)	Group Size	Enclosure Complexity (Species Specific e.g., Planting, Water Bodies, Wallows, Food, Shelter, Hiding Places etc.)	Nutrition	Accessibility (e.g., How Much of The Enclosure Can They Access)	Contingent Events (e.g., Visitor Events/Educational Aids, Building Work, Enclosure Changes, Animal Moves, Bin Collection, Deliveries)
1	Optimal species specific conditions Ideal Conditions-species-specific see Fiorito et al., 2015 Appendix 2B [21]	Enclosure mirrors the species’ wild habitat and is suitable for the species housed in terms of location, public viewing, proximity to other animals etc.	Group size in adherence with natural group size; stocking density appropriate for the enclosure; group structure is suitable	Enclosure complexity is equal to the wild environment. All natural behaviours can be expressed and there is no requirement for staff intervention e.g., additional enrichment	Nutrition provided optimally meets species specific and individual requirements (Nutritional, physiological and behavioural)	Access to all of enclosure	None
2	Slight variation from optimal conditions but still within 15% suggested range	Lacks one factor found in wild	Group structure differs slightly from a suitable group structure	All natural behaviours can be expressed with a minimal amount of staff intervention	Nutrition provided has minimal reduced suitability to meet species specific or individual requirements	Access in enclosure restricted by 25% for part of the day	External (to enclosure) event with minimal disruption
3	Slight variation from optimal conditions but still within 25% suggested range	Lacks two/three factors found in wild	Presence of more or less animals when compared with natural group size range, however, no overstocking	All natural behaviours can be expressed with considerable staff intervention	Nutrition provided has minimal reduced suitability to meet species specific and individual requirements	Access in enclosure restricted by 25% for one day	External event with mild disruption
4	Moderate variation from optimal condition with measurements within 25% from the edge of tolerance ranges	Lacks four/five factors found in wild	Group structure differs from a suitable group structure	Most natural behaviour can be expressed with minimal staff intervention	Nutrition provided has reduced suitability to species requirements but satisfies individual requirements	Access in enclosure restricted by 50% for part of the day	External event with some disruption OR enclosure furnishings changed with no other events taking place
5	Moderate variation from optimal conditions with measurements on the edge of tolerance range and recovery within 5 h	Lacks six factors found in wild	Stocking density slightly higher then appropriate for the enclosure (presence of many young without loss of the adults)	Most natural behaviours can be expressed with considerable staff intervention	Nutrition provided has reduced suitability to individual requirements but satisfies species requirements	Access in enclosure restricted by 50% for one day	External event with noticeable disruption OR movement into a familiar enclosure with no other events taking place
6	Significant variation from optimal conditions (outside of tolerance range) with recovery within 5 h	Lacks seven factors found in wild	Presence of more or less species than when compare with natural group size range, with slight overstocking	Some natural behaviours can be expressed with considerable staff intervention	Nutrition provided unsuitable to meet behavioural requirements of species and individual	Access in enclosure restricted by 75% for part of the day	External event causing noticeable disruption and movement into familiar enclosure OR interaction with public occurring in water
7	Significant variation from optimal conditions (outside of tolerance range)—not showing full recovery to normal or recovery taking over 5 h	Lacks eight factors found in wild	Group structure shows a large difference from a suitable group structure	Enclosure complexity and staff intervention are minimal, preventing the expression of numerous natural behaviours	Nutrition provided unsuitable to meet physiological requirements of species and individual	Access in enclosure restricted by 75% for one day	Movement into unfamiliar enclosure or introduction of new unfamiliar animal OR interaction with public occurring in water
8	Severe variation from optimal range with short term impact and expected recovery OR moderate to severe signs with long term impact on animals welfare and little chance of recovery	Lacks nine factors found in wild	Stocking density higher then appropriate for the enclosure	Enclosure complexity and staff intervention are minimal, preventing the expression of most natural behaviours	Nutrition provided unsuitable to meet behavioural and physiological requirements of species and individual	Access in enclosure restricted by 75% for > one day	External event causing definite disruption and movement into unfamiliar enclosure or introduction of new unfamiliar animal OR interaction with public occurring and handling out of water < 1 min
9	Severe variation from optimal range showing serious negative impact on the species ability to perform normal behaviours	Lacks ten factors found in wild	Stocking density higher then enclosure can support	Enclosure complexity and staff intervention is very limited, preventing the expression of almost all behaviours	Nutrition provided unsuitable to meet behavioural, physiological and nutritional requirements of species and individual	Removed from enclosure for part of the day	Movement into new unfamiliar enclosure and introduction of new animal(s) OR interaction with public occurring and handling out of water < 2 min
10	Lethal conditions with temperatures and chemical measurements far below/above suggested lethal range rendering the species recumbant/unable to carry out any normal behaviour	Lacks more than 10 factors found in wild	Large difference between natural group size and housed group size; or large degree of overstocking the enclosure	The options are not available in the enclosure nor provided additionally for the animal to express natural behaviours	No nutrition provided	Removed from enclosure for > one day	Combination of events: External prolonged event, movement into new unfamiliar enclosure, introduction of new unfamiliar animals. With extreme detrimental levels of disruption OR interaction with public occurring and handling out of water > 2 min

**Table 4:** Procedural parameter scoring for the AWAG assessment (Italics—factors assessed in cephalopods only).

Score	Isolation/Restraint	Effect of Intervention	Impact of Veterinary Procedures	Change in Daily Routine	*Sedation/Anaesthesia*
1	No Isolation/Restraint	No effect	No veterinary procedure	No change	*No sedation/anaesthesia*
2	Isolated for less than 3 h—Restrained for up to 2 min	Animal(s) carries out normal behaviour with no evidence of effect	Minor veterinary procedure carried out without difficulty. Minimal stress/effect on the animal(s) only lasting the length of the procedure	Animal(s) does not appear to notice any change	*Mild brief sedation with smooth induction and recovery. Quick return to natural feeding and behaviour*
3	Isolated for less than 6 h—Restrained for up to 15 min	Animal(s) shows mild stress behaviour as a response to the intervention but returns to normal as soon as the intervention is over	Minor veterinary procedure with some short term stress/effect on the animal(s). Recovery from stress <2 h	Animal(s) shows mild stress behaviour in response to change but returns to normal as soon as interaction is over	*Mild longer sedation with smooth induction and recovery. Quick return to natural feeding and behaviour*
4	Isolated for less than 12 h—Restrained for up to 30 min	Animal(s) shows mild stress behaviour in response to the change but takes upto 24 h to return to normal	Minor veterinary procedure with some medium term stress/effect on the animal(s).Recovery from stress <6 h	Animal(s) shows mild stress behaviour in response to change but takes up to 24 h to return to normal	*Sedation with stressful induction or recovery. But quick return to natural feeding and behaviour*
5	Isolated for less than 24 h—Restrained for up to 1 h	Animal(s) shows moderate stress behaviour in response to the change and takes more than 24 h to return to normal behaviour	Moderate veterinary procedure with noticeable short term stress/effect on the animal(s).Recovery from stress <6 h	Animal(s) shows moderate stress behaviour in response to the change and takes more than 24 h to return to normal behaviour	*Sedation with stressful induction and recovery. But quick return to natural feeding and behaviour*
6	Isolated for more than 24 h—Restrained for up to 2 h	Animal(s) shows moderate stress behaviour in response to the change and takes more than 48 h to return to normal behaviour	Moderate veterinary procedure with noticeable medium term stress/effect on the animal(s).Recovery from stress <12 h	Animal(s) shows moderate stress behaviour in response to the change and takes more than 48 h to return to normal behaviour	*Sedation with stressful induction and/or recovery. Short term effects on return to natural feeding and behaviour for few hours after procedure*
7	Isolated for more than 48 h—Restrained for up to 6 h	Animal(s) shows severe stress behaviour in response to the change but recovers quickly	Severe veterinary procedure with significant short term stress/effect on the animal(s). Recovery from stress >12 h	Animal(s) shows severe stress behaviour in response to the change but recovers quickly	*Sedation with stressful induction and/or recovery. Medium term effects on return to natural feeding and behaviour for more than 12 h after procedure*
8	Isolated for more than 1 week—Restrained for up to 12 h	Animal(s) shows severe stress behaviour in response to the change and takes more than 24 h to return to normal behaviour	Severe veterinary procedure with significant medium term stress/effect on the animal(s). Recovery from stress <24 h	Animal(s) shows severe stress behaviour in response to the change and takes more than 24 h to return to normal behaviour	*Sedation with stressful induction and/or recovery. Moderate to long term effects on return to natural feeding and behaviour for more than 48 h after procedure*
9	Isolated for more than 2 weeks—Restrained for up to 24 h	Animal(s) showing aggressive behaviour specifically following the change	Extensive procedure severely impacting the animal(s). Severe stress and/or aggressive behaviour displayed post procedure. Recovery from stress >24 h. Short term pain despite appropriate analgesia and treatment	Animal(s) showing aggressive behaviour specifically following the change	*Sedation with severely stressful induction and/or recovery. Long term effects on natural feeding and behaviour*
10	Isolated for more than 1 month—Restrained for more than 24 h	Animal(s) showing ongoing aggressive behaviour or harming itself as a result of the change	Extensive procedure severely impacting the animal(s). Severe stress and/or aggressive behaviour displayed for prolonged period post procedure. Recovery from stress >48 h. Long term pain despite appropriate analgesia and treatment	Animal(s) showing ongoing aggressive behaviour or harming itself as a result of the change	*Sedation with severely stressful induction and/or recovery. Prolonged effects on natural feeding and behaviour*

## Data Availability

Both adapted AWAG templates and all data gathered and used are available at: [38]-hNtlAf5abqx8aS7JZda?dl=0; Software used in this study is available at: https://zoo.awag.org.uk/ (accessed on 20 May 2022).
